# EcoDragons: A Game for Environmental Education and Public Outreach

**DOI:** 10.3390/insects12090776

**Published:** 2021-08-29

**Authors:** Rassim Khelifa, Hayat Mahdjoub

**Affiliations:** 1 Zoology Department, University of British Columbia, Vancouver, BC V6T 1Z4, Canada; 2 Biodiversity Research Centre, University of British Columbia, Vancouver, BC V6T 1Z4, Canada; 3 Department of Evolutionary Biology and Environmental Studies, University of Zurich, Winterthurerstrasse 190, 8057 Zürich, Switzerland; hayatmahdjoub@gmail.com

**Keywords:** dragonfly, ecology, conservation, community science, pedagogy

## Abstract

**Simple Summary:**

We face serious ecological and societal issues that require a rethinking of our approaches to involving the public in problem-solving and decision-making. Researchers need help from community scientists to gather data and generate fruitful discussions to tackle planetary problems. However, the establishment and maintenance of strong links between scientists and society require innovative ways of communication outside conventional educational institutions. Here, we propose a game that teaches the players the basics of ecological thinking when approaching environmental issues and biodiversity conservation. The game is called EcoDragons and it uses dragonflies as the main biological entity to colonize, establish and maintain biodiversity in an empty landscape that regularly faces climatic and anthropogenic disturbances. While the current EcoDragons was based on European dragonflies, the concept is highly adaptable to dragonflies of other regions (changing the species names), or even to other taxonomic groups. Besides the various pedagogical benefits, the game has the potential to foster public engagement in biodiversity conservation and community science.

**Abstract:**

Environmental education is crucial to tackling the pressing ecological and societal issues on our planet. Although there are various ways to approach environmental education and raise public awareness, games are potentially an effective vehicle of knowledge and engagement because they vulgarize the scientific information in a universal ‘language’ and bring people together. Here, we designed a game, EcoDragons, that integrates principles of ecology, biological conservation, life history, and taxonomy. The protagonists of the game are dragonflies and damselflies. The aim of the game is to colonize habitats with different species and use ecological processes (e.g., predation, competition, and mutualism) and conservation measures (e.g., restoration and reintroduction) to face random environmental disturbances (e.g., climate warming, drought, pollution, and biological invasion). The version of the game presented in this paper was based on European species. The game includes 50 species (25 dragonflies and 25 damselflies). The winner of the game is the one who occupies more habitats, establishes and maintains the largest number of species, and solves more anthropogenic disturbances. EcoDragons has a global outreach potential to educate the public about ecology, conservation, and organismic life history, and will probably engage people in environmental advocacy.

## 1. Introduction

Public outreach is an essential initiative to reach the ultimate goal of conservation biology and face environmental and societal issues [1]. Although public engagement has a positive effect on biodiversity conservation and environmental advocacy [2,3], it is often challenging to effectively build a bridge between researchers and the public [4], particularly with young students who will play a pivotal role in future behavioral decisions towards the environment and policymaking. The main challenge is the difficulty to find a way to vulgarize the information and transfer knowledge outside formal learning environments using other means than scientific papers and textbooks [5]. One of the most effective ways to attract, retain, and engage the public in research and environmental awareness is games [6,7].

Social games have been played by people for thousands of years [8]. They are not only a way to spend leisure time and reduce stress but also an opportunity to promote creativity, learn new skills, train the mind, and create social relationships [9]. Unlike science, which is dominantly unilingual, a game is a ‘universal language’ that transcends cultural and linguistic barriers. Thus, integrating games in environmental education is a powerful initiative to facilitate the communication of complex scientific knowledge to a large audience of different ages and backgrounds [10]. The principles of ecology and conservation offer many opportunities to develop game concepts [11] that communicate many of the “must-know” concepts that the general public should be aware of [12]. For instance, the relationship of species with their environment, the importance of ecological and evolutionary processes to maintain biodiversity, and the need for conservation actions to protect natural environments and species against anthropogenic challenges are all aspects that improve ecological literacy and increase awareness of the public about the current and future environmental issues. 

Here we present a game, EcoDragons, that educates players about the basic principles of ecology and conservation from the perspective of dragonflies (Insecta: Odonata). The game offers 133 cards of three categories (Table 1) and a landscape with five aquatic habitat types. It includes species cards (50 species), action cards (ecological, evolutionary, and conservation processes), and anthropogenic disturbance cards (climate change, invasive species, and habitat fragmentation). The aim of the game is to colonize empty habitats with species, increase species diversity, and solve randomly arising environmental disturbance with various action cards that involve ecological and evolutionary processes as well as different conservation measures.

## 2. The Protagonists—Dragonflies

Dragonflies are widespread aquatic insects that live in most continents except the Antarctic. Dragonflies belong to the order Odonata which is mainly divided into three existing suborders: *Zygoptera* (damselflies), *Anisoptera* (dragonflies), and *Anisozygoptera*. There are about 7000 species worldwide that live in various freshwater systems, including both running and standing water [13]. They have a complex life cycle with an aquatic egg and larval stage and a terrestrial adult stage. In the temperate region, adults are most active during spring and summer [14]. The adults are predators of smaller insects and other arthropods, including agricultural pests and transmitters of human disease [15]. Dragonflies are also eaten by a large array of animals, including arthropods, amphibians, and birds. Thus, they play a crucial role in ecosystem functioning and human well-being. They have been studied for more than a century [16], and are extensively used in ecological and evolutionary research [17]. For instance, their sensitivity and rapid response to warming and environmental disturbance make them a good barometer of climate change and anthropogenic disturbance [18]. Dragonflies are charismatic, fascinating, not harmful to humans, and appealing to the public, which promotes their potential for long-term community science projects and public outreach programs [19]. Here, dragonflies of Europe were selected as the protagonist of the game [20,21], but other versions of the game including Western Canadian (British Columbia) and North African odonates are in development. For each region, we selected only a subset of species (here, 50 species). Table S1 presents the list of the 50 species used for European dragonflies.

## 3. Game Objective and Rules

The objective of the game is to occupy as many habitats as possible and maintain biodiversity in the environment using different ecological, biological, and evolutionary processes as well as conservation measures to face anthropogenic disturbance. The game can be played by two people or more (ideally 2–4 persons). The board represents the landscape (environment) which includes five habitat types (lake, pond, marsh, river, and stream). Besides the landscape, three types of cards are provided in three separate decks, namely dragonfly, action, and human disturbance cards. Dragonfly deck includes all species of odonates that should be used to colonize habitats by placing them in their preferred habitat. After the use of all dragonfly cards the game stops. The action deck includes ecology cards, conservation cards, and bonus cards; all of which play a particular role to protect species and habitat or eliminate the opponent species using biotic interactions. The anthropogenic disturbance deck includes different sources of human impact on the environment which reduce habitat quality and threaten the persistence of dragonflies. 

At the beginning of the game, each player receives five cards: three dragonfly cards and two action cards. In each round, each player must draw a card from one of the three decks. To draw a card, the player must roll a dice to determine which deck should be used to draw a card. The six-sided die has two sides for each of the three categories of cards. After drawing a card, the player can either play a card or pass. Playing a card means placing it on a specific habitat type (one of the five types). However, if the card is an anthropogenic disturbance, it is immediately placed in the indicated habitat type, thus mimicking a random environmental disturbance action. Each habitat can be occupied by many species and each species has specific habitat preferences. Thus, habitats are gradually occupied by dragonfly cards and occasionally affected by habitat disturbance. The players should actively avoid the decline of habitat quality induced by anthropogenic factors, protect species from extinction, and restore habitats if they are disturbed.

At the end of the game (when the dragonfly deck is depleted), players calculate the total score based on the number of habitats occupied, the number of disturbances solved, and the sum of conservation priority indices of each dragonfly. A description of the gameplay of EcoDragons is given in Supplimentary Material File S1.

## 4. Environment 

**Figure 1:**
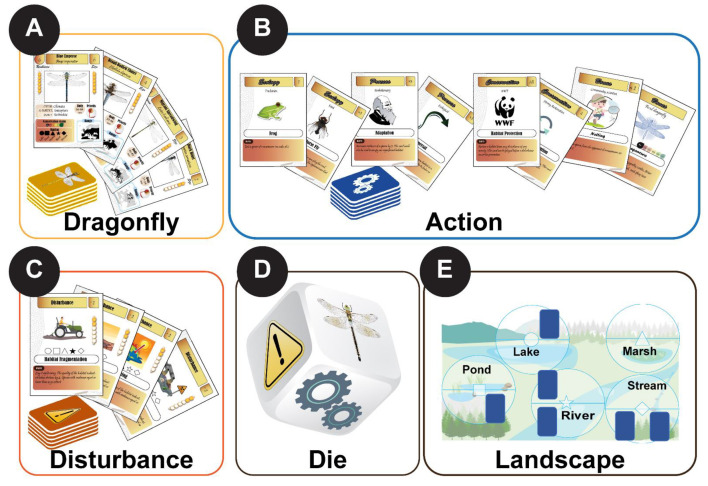
The components of EcoDragons. Three types of cards are provided: (**A**) dragonfly, (**B**) environmental disturbance, (**C**) action cards (ecology, conservation, process, and bonus cards). The cards are separated into three decks. (**D**) A die is used to determine which deck should be used to draw cards. (**E**) The play environment includes five habitat types (lake [circle], pond [square], marsh [triangle], river [star], and stream [diamond]).

The game starts with an environment presenting five empty habitat types (lake [circle], pond [square], marsh [triangle], river [star], and stream [diamond]) (Figure 1). The players could decide whether habitats should have a maximum carrying capacity (K) or not. In this game, K is a number that corresponds to the sum of size indices of species. For instance, the large blue imperor (*Anax imperator*) has a size index of 6 whereas the small blue-tailed damselfly (*Ischnura elegans*) has a size index of 1. Thus, if K is 20, the players could only occupy the habitat with a set of species whose sum of size index ≤ 20, such as species 1 [size index = 6] + species 2 [5] + species 3 [4] + species 4 [3] + species 5 [2]. K of each habitat could be randomly attributed with a roll of a die (e.g., one is equivalent to a total size index 10 and six is equivalent to a total size index 60). 

Pedagogical relevance: Here, the player learns to differentiate between the physical and biotic aspects of the habitat which is usually colonized by multiple species that live in assemblages. The player will also learn that species have particular habitat preferences and each habitat could harbor a limited number of species, its so-called carrying capacity. Additionally, players should understand that the establishment of high biodiversity depends on the availability of heterogeneity of habitats in the landscape.

## 5. Cards 

EcoDragons includes three elements: an environment with five habitat types (wetlands), cards with six categories, and a die for random processes. The number of cards of each category is shown in Table 1 and all cards are shown in Supplimentary Material File S2.

### 5.1. Species Cards

**Figure 2:**
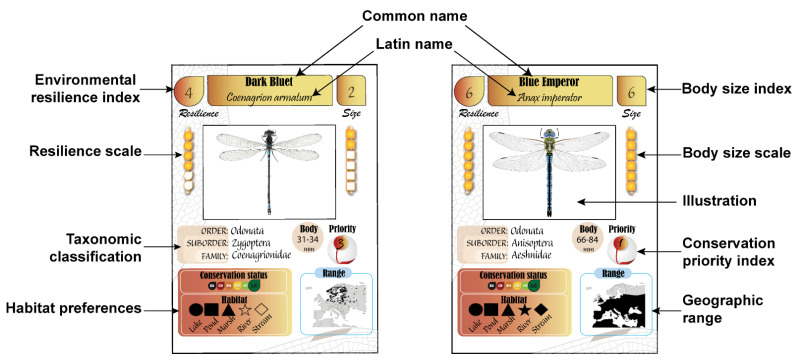
Details included in the species card of EcoDragons.

There is a total of 50 species (25 damselflies and 25 dragonflies), belonging to 9 families and 21 genera (Table S1). There is high body size variability among species; with a wingspan ranging between 25 mm in *Ischnura* damselfly and 110 mm in *Anax* dragonflies. In the game, each species is represented in a unique card that indicates different information, including a body size index, environmental resilience index, taxonomic classification, habitat preferences, conservation status, a conservation priority index, the body length in mm, a geographic distribution, and an illustration of the male adult (Figure 2). In each of these parameters, there is high interspecific variability, which often corresponds to the natural variability. Below is a description of each component.

- A body size index ranges between one and six, where one indicates a small size (e.g., *Ischnura*) and six indicates a large size (e.g., *Anax*). The body length in mm is also provided based on Dijkstra and Schröter [21].
- An environmental resilience index also ranges between one and six, where one indicates low resilience (meaning high sensitivity to environmental disturbance), and six indicates high resilience.
- Taxonomic classification shows the order, the suborder, and the family name. The common and Latin name of species is also given at the top of the card.
- Habitat preferences include lakes, ponds, marshes, rivers, and streams, indicated by the following symbols: triangle, square, circle, star, and diamond. Some species are habitat generalists and live in multiple habitat types (e.g., *Pyrrhosoma nymphula* which lives in various types of wetlands) whereas other species are habitat specialists and live in one or two habitats (e.g., *Calopteryx splendens* lives in rivers and streams).
- Conservation status is based on the European IUCN Red List and includes the following ranks: Least Concern (LC), Near Threatened (NT), Vulnerable (VU), Endangered (EN), Critically Endangered (CR), and Extinct (EX). A threatened species (NT, VU, EN, and CR) receives an extra score (conservation status score). LC, NT, VU, EN, and CR give 1, 2, 3, 4, and 5 points, respectively.
- Conservation priority index (CPI) reflects whether a species should receive special conservation attention. This index is very important in the game because it counts in the total score at the end of the game. It is calculated as: CPI = (6 − resilience index) + conservation status score. For example, *Anax imperator* has a resilience index of 6 and is ranked LC (conservation status score = 1), thus CPI = 6 − 6 + 1 = 1. However, *Lestes macrostigma* has a resilience index of 3 and is ranked VU (conservation status score = 3), thus CPI = 6 − 4 + 3 = 5. Thus, *L. macrostigma* has a higher conservation priority than *A. imperator*.
- Geographic distribution is illustrated with a map that includes Europe and part of North Africa and Western Asia. The limits of the map were fixed to 31° N–72° N latitude and 17° W–44° E longitude. Most maps were produced using shapefiles obtained from the IUCN red list website (https://www.iucnredlist.org/, accessed on 28 July 2021), R packages tmap [22], and sf [23]. When the shapefiles were not found, maps were produced manually based on Dijkstra and Schröter [21].
- Pedagogical relevance: Players are exposed to a large number of basic facts about dragonflies. Players learn that there are interspecific differences in body size, environmental sensitivities, conservation status, geographic range, and habitat preferences in a single assemblage. They also learn that some species are habitat specialists and thus are more threatened to extinction than habitat generalists. Further, some species of conservation concern have a small range size and thus need particular conservation attention. The brief taxonomic classification allows the players to learn the major taxonomic ranks and the name of families and species.

### 5.2. Anthropogenic Cards

**Figure 3:**
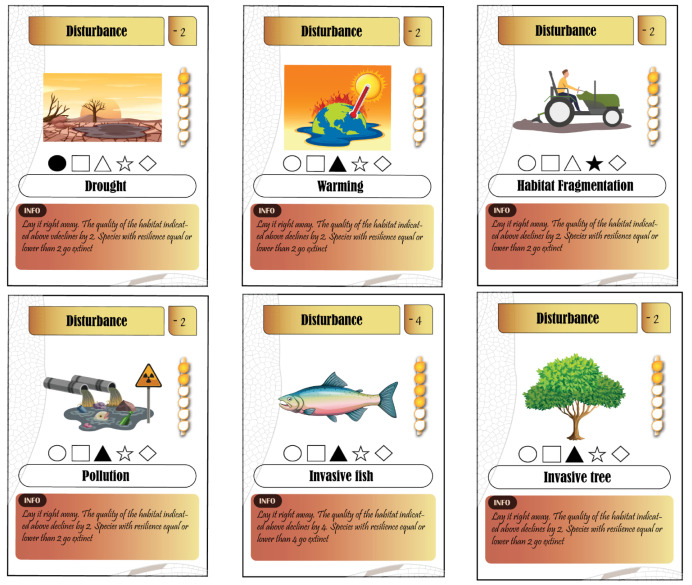
Types of anthropogenic cards.

The game provides three types of anthropogenic disturbance in six cards: (1) Climate change includes drought and warming, (2) habitat degradation includes habitat fragmentation and pollution, and (3) biological invasion includes invasive fish and invasive trees. Because the draw of the cards is determined with a roll of a dice, anthropogenic cards mimic a random process of environmental disturbance. Each card has three levels of severity and is intended for a specific habitat type (Figure 3). Anthropogenic cards reduce the initial base level quality of habitat by 2 (mild), 4 (quite strong), or 6 points (severe). Depending on the severity of the anthropogenic disturbance and the resilience index of species, species could go extinct or survive. For example, if a habitat harbors two species with a resilience index of 3 and 5 and the anthropogenic disturbance severity is 4, the species with a resilience index of 5 survives whereas that of a resilience index of 3 goes extinct. Anthropogenic cards have a permanent impact on habitats if they are not countered with a conservation card. 

Pedagogical relevance: Players get to know different anthropogenic disturbances and their negative impact on biodiversity and freshwater systems. They will also understand the permanent aspect of anthropogenic disturbance and the need for human intervention to restore habitats and protect species. Anthropogenic cards also highlight that some species are more prone to extinction than others due to their habitat specialization and resilience to environmental disturbances. 

### 5.3. Action Cards

This category of cards allows the players to perform different types of actions to maintain dragonfly diversity, protect habitats, avoid anthropogenic disturbance, and increase the score. Action cards include different groups of cards: ecology, conservation, process, and bonus cards.

#### 5.3.1. Ecology Cards

**Figure 4:**
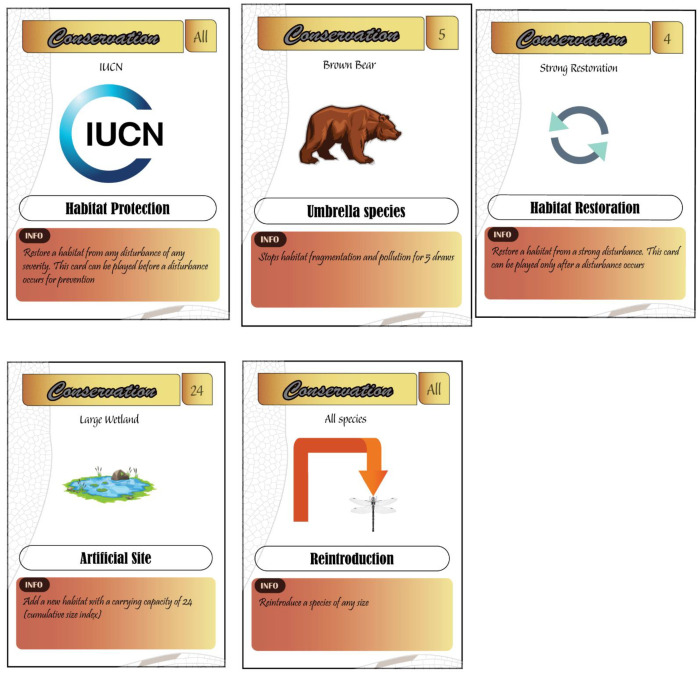
Types of conservation cards.

Different ecological processes are included in this game (Figure 4). Ecological factors such as abiotic (weather conditions) and biotic factors (predation, intraguild predation, competition, parasite, and mutualism) are provided.

- Weather: In nature, dragonflies are active in warm and sunny weather, and adverse weather conditions such as rain, wind, and frost stop their activity. To reflect this effect in the game, abiotic cards stop the player from playing for one (wind and rain) or two rounds (frost).
- Predation: Three predators with different size-dependent predation capacities are provided (frog, passerine, and hawk). The frog eats only species with size indices ≤2. The passerine bird eats species with size indices ≤4, whereas the hawk eats only large dragonflies ≥5. While this predator-prey interaction might not accurately reflect the interactions in the real world (e.g. a frog or a passerine could eat a large dragonfly), it introduces more complexity in the game and highlight the potential size-dependent structure in community interactions.
- Intraguild predation: This kind of predation is also a process by which a dragonfly species could eat a smaller or equally sized species living in the same habitat.
- Competition: This refers only to interspecific competition. If players share the same habitat, competition can be used to exclude a species of a smaller size.
- Parasitism: Three levels of parasitism intensity are provided. Parasitism reduces the resilience of species by 1, 2, or 3 points. Parasites could also be used to exclude an opponent dragonfly from a habitat if the resilience is reduced to 0.
- Mutualism: This interaction benefits different species living in the same habitat. There are three levels of mutualism depending on the number of families. If the species belong to the same family, the resilience increases by 1, two families increase resilience by 2, and so on. Mutualism allows the player to find combinations of species to improve the resilience of assemblages to anthropogenic disturbance.
- Resource: Resource cards include three types of food (fruit fly, mosquito, and horsefly) that dragonflies usually eat. These cards increase the resilience of species to environmental disturbance.

Pedagogical relevance: Players learn the major ecological forces that benefit and affect species in their natural habitats. Ecology cards help players to differentiate between abiotic and biotic factors, comprehend how species interact with each other, and understand the importance of diversity in increasing the resilience of species. They also teach players that dragonflies are both prey and predators in their natural habitat, and thus they play a crucial role in the foodweb of freshwater ecosystems.

#### 5.3.2. Conservation Cards 

**Figure 5:**
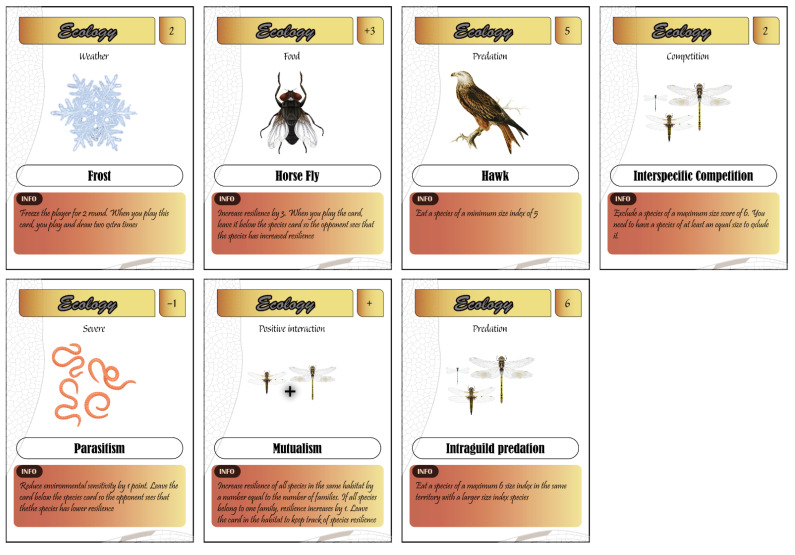
Types of ecology cards.

There are different conservation measures provided in this game to prevent habitat degradation or counter anthropogenic disturbance. Conservation cards include international organizations, habitat restoration, umbrella species, reintroduction, and artificial site creation (Figure 5).

- Conservation organizations (IUCN, WWF, Ramsar, Wildlife Conservation Society, Conservation International, and Nature Conservancy) are used to protect habitats (prevention) or stop any type of existing anthropogenic disturbance of any severity.
- Habitat restoration cards have three levels of effectiveness to reduce the severity of anthropogenic disturbance. They can be played only when the habitat is disturbed.
- Reintroduction is another conservation measure that players could perform in the game to reintroduce a species that went extinct by any source or mortality.
- Umbrella species allow players to stop habitats from being impacted by specific types of anthropogenic disturbance (pollution, biological invasion, and habitat fragmentation) for a number of draws, which depend on the type of umbrella species. Umbrella mammals (brown bear and European mink) protect the habitat for five draws, umbrella birds (red-breasted goose and aquatic warbler) protect the habitat for four draws, and umbrella amphibians (fire salamander) and reptiles (European pond turtle) protect the habitat for three draws. Note that the umbrella species used in the game are threatened aquatic vertebrates in Europe.
- Artificial site cards allow the player to provide a new habitat for species to either increase the carrying capacity or to provide a refuge site for species that were excluded by anthropogenic disturbance. An artificial site card has three levels with different carrying capacities. The player has to specify which kind of habitat the artificial site is when played.

Pedagogical relevance: The player is exposed to important topics in conservation biology. First, they will learn that conservation organizations are crucial to ensure the persistence of species in their natural habitat in a rapidly changing world. The players learn the importance of habitat restoration and species reintroduction as crucial practices in case of an important environmental disturbance or population extirpation. They also learn the concept of umbrella species and the ecological benefits of conserving a single large species (often vertebrates) to smaller species. In addition, creating artificial sites to face environmental disturbance is also a topic that players will understand and incorporate into their arsenal of potential sustainable solutions [24]. 

#### 5.3.3. Process Cards

**Figure 6:**
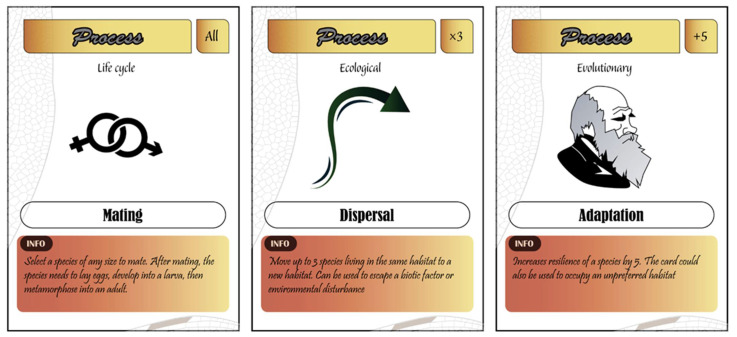
Types of process cards.

There are three types of processes provided: biological (four cards), evolutionary (one card), and ecological (one card) (Figure 6).

- The ecological process provided is dispersal, which allows the player to escape anthropogenic disturbances and biotic attacks. When used, a species needs to find an adequate type of habitat to disperse to, otherwise, it will not survive.
- Evolutionary processes include adaptation to a new habitat (occupy a habitat where initially the species does not prefer) or to anthropogenic and biotic disturbance. For instance, if drought severity surpasses the dragonfly’s resilience, adaptation can be used to increase species resilience.
- Biological processes represent the life cycle of species, including mating, egg-laying, larva, and metamorphosis. The player has to collect and sequentially or simultaneously play the four cards to duplicate a species (a dragonfly card will count as double). When the species is killed after egg deposition (by predation, parasitism, or competition), the adult dies, but the egg remains and could be developed to the adult stage. If the duplicated species card stays until the end of the game, the player scores twice the number of points for the species card.

Pedagogical relevance: This category of cards helps the player to understand basic ecological, evolutionary, and biological mechanisms that species use to escape environmental disturbances. Dispersal and adaptation have a pivotal role in the maintenance of populations and species in their natural environment, and thus are important drivers of biodiversity. Through the sequential reproductive process of cards, the player learns the life cycle of dragonflies and understands that species have a complex life cycle with an aquatic egg and larval stage and a terrestrial adult stage. The player also realizes that survival from the mating stage to the emergence of the species is a lengthy process, which is often the case in dragonflies.

#### 5.3.4. Bonus Cards

**Figure 7:**
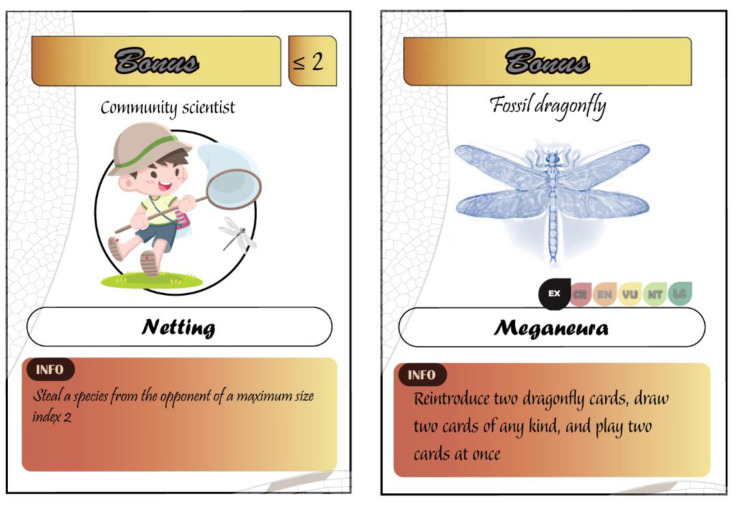
Types of bonus cards.

Besides the types of cards discussed above, there are five bonus cards that increase the multidimensionality of the game and the intellectual knowledge of the players (Figure 7).

- Netting (trapping) cards are used to steal a species from the opponent. They feature three levels of collectors (community scientists, students, and an expert odonatologist) that have different abilities to trap species of different sizes. For instance, a community scientist (a kid) can only catch damselflies, whereas an expert odonatologist can catch any species.
- Fossil species: The game includes two supplementary fossil dragonfly cards that confer special abilities to the player. First, the giant fossil dragonfly called *Meganeura* (the largest known dragonfly with about 70 cm of wingspan). Second, Cephalozygoptera is a recently discovered fossil suborder in the order Odonata [25]. Both cards allow the player to reintroduce two dragonfly cards, draw two cards of any kind, and play two cards at once.

Pedagogical relevance: This category of cards raises the awareness of the player on community science and the importance of data collection for the understanding of biodiversity. The player will also learn about some key groups of dragonflies (fossil) that used to exist but are currently extinct, highlighting the existence of the extinction process and the probable exacerbation of its rate by human activity.

## 6. Relevance to Environmental Education and Public Outreach

Science communication to the public is essential to reinforce the link between scientists and the general public and strengthen scientific impact [26]. EcoDragons has an important potential for environmental education and public outreach for climate change, anthropogenic disturbance, and biodiversity conservation (Table 2). The game offers a wealth of information on the fields of ecology, evolution, conservation, and entomology, and thus is a playful medium of essential concepts in environmental thinking that could reach a large audience, including youngsters. EcoDragons is likely an alternative way to face misconceptions and misinformation about insects [27]. Thus, the game presents an opportunity to train the next generation from an early age to think about environmental issues, develop a sense of responsibility towards the environment and biodiversity, and shift individual behavior to be more environmentally friendly. Because the game could be played by two to four players, it has the potential to disseminate among people through social interactions and consequently could expand the pedagogical messages across people. Moreover, EcoDragons could transfer the same uniform information to different people from different countries, cultures, and languages, and thus offers a global outreach potential [28]. Additionally, the game could be adapted to the biodiversity of different regions worldwide (e.g., the current list of dragonflies of Europe could be changed with those of South America, southwest Asia, or North Africa) to promote environmental education, popularize key taxonomic groups, and foster environmental advocacy for local biodiversity. While EcoDragons was based on dragonflies, the concept is highly adaptable to other taxonomic groups of insects, amphibians, reptiles, birds, and mammals. Finally, players of EcoDragons could develop an emotional relationship with the protagonists (taxonomic group), which might lead them to engage in community science, biodiversity conservation, and environmental advocacy [29].

## 7. Future Developments

Ideally, a digital version of EcoDragons will be developed and made available online for global outreach. Video game development is a great interdisciplinary opportunity to bring scientists, game developers, and artists together to collaborate on ecological education and public outreach. The video game will gradually include more and more species from different regions worldwide and will eventually provide the majority of described odonates of the world. This will allow players to select the odonates of specific geographic regions where they would like to play and thereby learn the different odonate assemblages of the world. Furthermore, the online video game will open many possibilities for improvement and new implementations. On one hand, the gaming community could share their experience and make suggestions regarding the rules and gameplay to ameliorate the player’s experience. On the other hand, the scientific community could also suggest new ecological, evolutionary, and conservation concepts to implement in the game to increase the educational value and environmental literacy. 

## Figures and Tables

**Table 1:** The number of cards of each category of EcoDragons.

Card Category	Number of Components	Replicates (Card Levels)	Total Number of Cards
Species	50	1	50
Disturbance	6	3	18
Action (Ecology)	7	3	21
Action (Conservation)	7	3	21
Action (Process)	6	3	18
Action (Bonus)	2	2 & 3	5
Total	78		133

**Table 2:** Content and pedagogical relevance of the different elements and types of cards in EcoDragons.

Element	Type	Contents	Pedagogical Relevances
**Environment**	Aquatic	Habitats Carrying capacity	Diversity of habitats in nature Limits to the capacity of habitats to welcome species
**Cards**	Species	Taxonomic classification: Suborder, family, common and Latin species Ecology: Environmental resilience, habitat preferences, geographic distribution Biology: Body size and behavior	General taxonomy Dragonfly identification Interspecific variability in body size, ecology, and conservation threat
	Action (Ecology)	Abiotic factors: heat, cold, and wind Biotic factors: predation, intraguild predation, competition, parasitism, and mutualism	Dragonfly activity is highly dependent on the weather. Dragonflies are not active in bad weather Basic essential knowledge in the various types of ecological factors. Ecological barriers to species persistence Importance of species interactions in structuring communities
	Action (Conservation)	Conservation organizations: WWF, IUCN, Nature Conservancy, Ramsar Conservation measures: habitat restoration, species reintroduction, umbrella species; artificial sites	Non-profit international organizations for biodiversity conservation Understanding the different methods used to cope with biodiversity loss The importance of the conservation of larger species for smaller species The realization that most environmental problems are anthropogenic
	Action (Processes)	Ecological process: Dispersal *Evolutionary process*: Adaptation Biological process: Life cycle (copulation, eggs, larva, metamorphosis, and adult)	Essential knowledge of ecological, evolutionary, and biological strategies that species use to cope with environmental change Environment, evolution and demography are species’ toolkits to persist in a changing world Dispersal connects communities and shapes habitat biodiversity
	Action (Bonus)	Traping: Community scientists, students, and odonatologist Fossil dragonfly: *Meganeura* and Cephalozygoptera	Disseminating the role of community science in data collection and engagement for research Some of the ancient dragonflies were huge New taxonomic discoveries showed an extinct group (suborder) of dragonflies Awareness of the extinction process which could be exacerbated with human activity
	Anthropogenic disturbance	Climate change (drought and warming) Habitat degradation (habitat fragmentation and pollution) Invasive species (fish and tree)	Understanding of the negative impacts of anthropogenic factors on species and assemblages. Awareness that while anthropogenic impacts can permanently damage natural habitats, they could also be reversible.
	Die	Random process	Stochasticity plays an important role in determining the fate of populations and species.

## Data Availability

The data presented in this study are openly available in supplementary material.

## Supplementary Materials

The following are available online at https://www.mdpi.com/article/10.3390/insects12090776/s1. supplementary material File S1: A sample of gameplay of EcoDragons, supplementary material File S2: All cards used in EcoDragons, Table S1: Species list of European species and all other cards used to design EcoDragons.
